# Grayscale medical image segmentation method based on 2D&3D object detection with deep learning

**DOI:** 10.1186/s12880-022-00760-2

**Published:** 2022-02-27

**Authors:** Yunfei Ge, Qing Zhang, Yuantao Sun, Yidong Shen, Xijiong Wang

**Affiliations:** 1 School of Mechanical Engineering, Tongji University, Shanghai, China; 2 Department of Orthopaedics, The First People’s Hospital of Yancheng, Yancheng, China; 3 Shanghai Bojin Electric Instrument and Device Co., Ltd, Shanghai, China

**Keywords:** Grayscale medical image, Image segmentation, Deep learning, Object detection, Point cloud

## Abstract

**Background:**

Grayscale medical image segmentation is the key step in clinical computer-aided diagnosis. Model-driven and data-driven image segmentation methods are widely used for their less computational complexity and more accurate feature extraction. However, model-driven methods like thresholding usually suffer from wrong segmentation and noises regions because different grayscale images have distinct intensity distribution property thus pre-processing is always demanded. While data-driven methods with deep learning like encoder-decoder networks always are always accompanied by complex architectures which require amounts of training data.

**Methods:**

Combining thresholding method and deep learning, this paper presents a novel method by using 2D&3D object detection technologies. First, interest regions contain segmented object are determined with fine-tuning 2D object detection network. Then, pixels in cropped images are turned as point cloud according to their positions and grayscale values. Finally, 3D object detection network is applied to obtain bounding boxes with target points and boxes’ bottoms and tops represent thresholding values for segmentation. After projecting to 2D images, these target points could composite the segmented object.

**Results:**

Three groups of grayscale medical images are used to evaluate the proposed image segmentation method. We obtain the IoU (DSC) scores of 0.92 (0.96), 0.88 (0.94) and 0.94 (0.94) for segmentation accuracy on different datasets respectively. Also, compared with five state of the arts and clinically performed well models, our method achieves higher scores and better performance.

**Conclusions:**

The prominent segmentation results demonstrate that the built method based on 2D&3D object detection with deep learning is workable and promising for segmentation task of grayscale medical images.

## Background

Medical imaging plays the key role in diagnosis or disease treatment by revealing internal structures with technologies mainly of computer tomography (CT), magnetic resonance imaging (MRI), ultrasound, and especially X-ray radiography [1]. Due to different absorption capability of various organs or tissues for radiations, waves, and etc., pixels belong to various object in grayscale medical images have diverse grayscale values usually from 0 to 255 [2] and meanwhile values of pixels of the same object always gather within a range.

Medical image segmentation has been widely applied to make images clearer with anatomical or pathological structures changes [3], such as bone segmentation [4], lung segmentation [5, 6], heart fat segmentation [7], liver or liver-tumor segmentation [8, 9] and Intracranial hemorrhage segmentation [10, 11], etc. They could be considered to divide origin images into several sub regions for picking up some crucial objects and extracting interesting features which improve the computer aided diagnostic efficiency. There has raised enormous approaches and they could be classified into two categories: model-driven techniques and data-driven techniques [5, 12].

Many model-driven methods for medical image segmentation, including thresholding, clustering, and region growing, were presented in particular before the widespread application of deep learning [12]. Thresholding was one of the most common used method in practice due to its efficiency [13]. The basic working of thresholding was to determine specific threshold values and each pixel in the image could be classified as the foreground or background depending on the comparison between their intensity values and threshold values [14–16]. Traditional thresholding methods always relied on single models for universal segmentation tasks which could lead to incorrect results. Also, segmentation objects often occupied only parts of whole images and pixels of different objects may share same intensity values, so noises could appear if image segmentation was applied overall.

With the era of big data coming, emerging data-driven technologies with deep learning have remarkably demonstrated in variety medical image segmentation task. Supervised learning methods and especially some convolutional neural network (CNN) based encoder-decoder structures such as fully convolutional networks (FCN) [17], U-Net [18], DeepLab [19] has practically proved [5]. Compared with traditional methods, deep learning could help analyze medical images more effectively and extract more detailed features.

Although these end-to-end structures was pragmatic for medical images semantic segmentation, the segmentation accuracy always relied on a large amount of training dataset. But medical image annotation could be time-consuming and quite expensive, thus transfer learning was used to solve the problem of limited labeled data and pre-trained networks on natural images as ImageNet [20] were often adopted for image segmentation [21, 22]. However, considering these datasets were mainly designed to train models for object detection or classification, they may be more suitable to pretrain networks for object detection. This inspired us to segment images with object detection. We find that grayscale images could be segmented according to the comparison of thresholding values with values of pixels in images and these pixels could be turned into 3D point cloud according to their positions and grayscale values. Thus, by applying 3D object detection in the point cloud, we could achieve groups of points within 3D bounding boxes. The top and the bottom of boxes represent the thresholding values for segmentation and after mapping these points into 2D images, corresponding pixels could compose segmented results. Besides, 2D object detection could determinate regions of interest (ROI) in grayscale medical images to reduce noises. Therefore, according to above strategy, we propose the grayscale medical image segmentation method based on 2D&3D object detection.

The remainder of this paper is organized as following: second section introduces the applied medical Image datasets and describes details of proposed technologies, while in third section the obtained results are displayed and the discussion is provided. Finally, forth section presents the conclusions as well as future work suggestions.

## Methods

### Image datasets

**Fig. 1:**
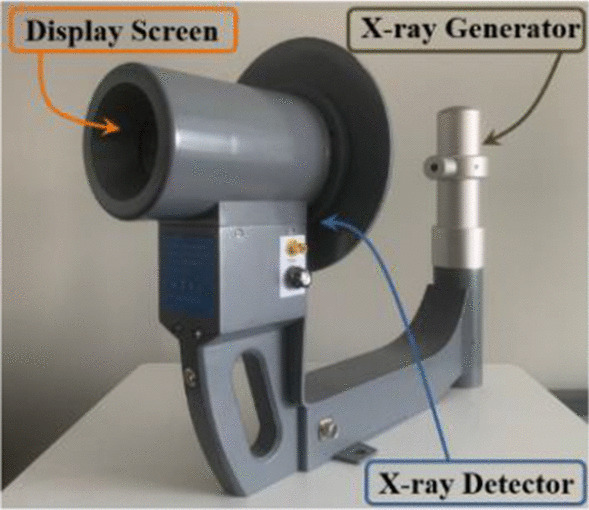
The portable X-ray machine

Since bone and chest X-ray images are the most common grayscale medical images in clinical, two typical sets of available datasets are prepared including musculoskeletal radiographs, and chest radiographs. Musculoskeletal radiographs about upper and lower extremity includes musculoskeletal radiographs (MURA) [23], lower extremity radiographs (LERA) [24] and prepared phalanx and forearm X-ray images. Chest radiographs mainly come from chest radiography (CheXpert) [25, 26]. MURA and LERA are large datasets of bone radiographs from Stanford University and they contain X-ray images about the upper and lower extremity respectively. CheXpert is a large dataset of chest radiographs and it is also from Stanford University. Besides, phalanx and forearm X-ray images are obtained with the portable X-ray machine as Fig. 1 Shown. Totally, 2509 cases among MURA and LERA, 3100 cases of CheXpert and 500 phalanx and forearm X-ray images are adopted for models training and validation.

**Fig. 2:**
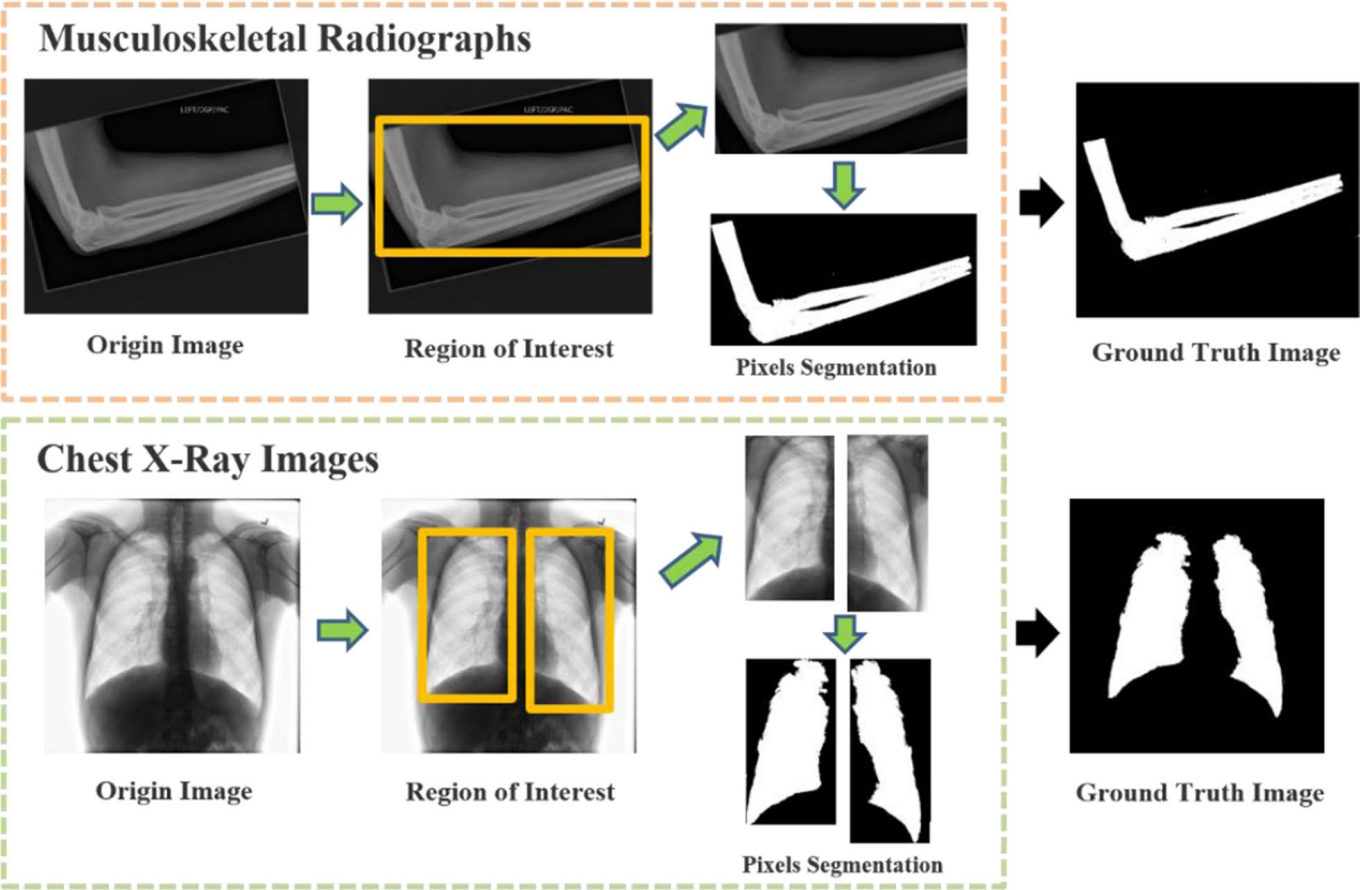
Examples of medical images in two datasets and manual segmentation results

The proposed grayscale medical image segmentation method is based on the supervised artificial intelligence techniques, and labels are performed manually in two types medical images for model training. Figure 2 shows origin images, and their respective Ground Truth (GT) images in different datasets.

### Grayscale image segmentation framework

**Fig. 3:**
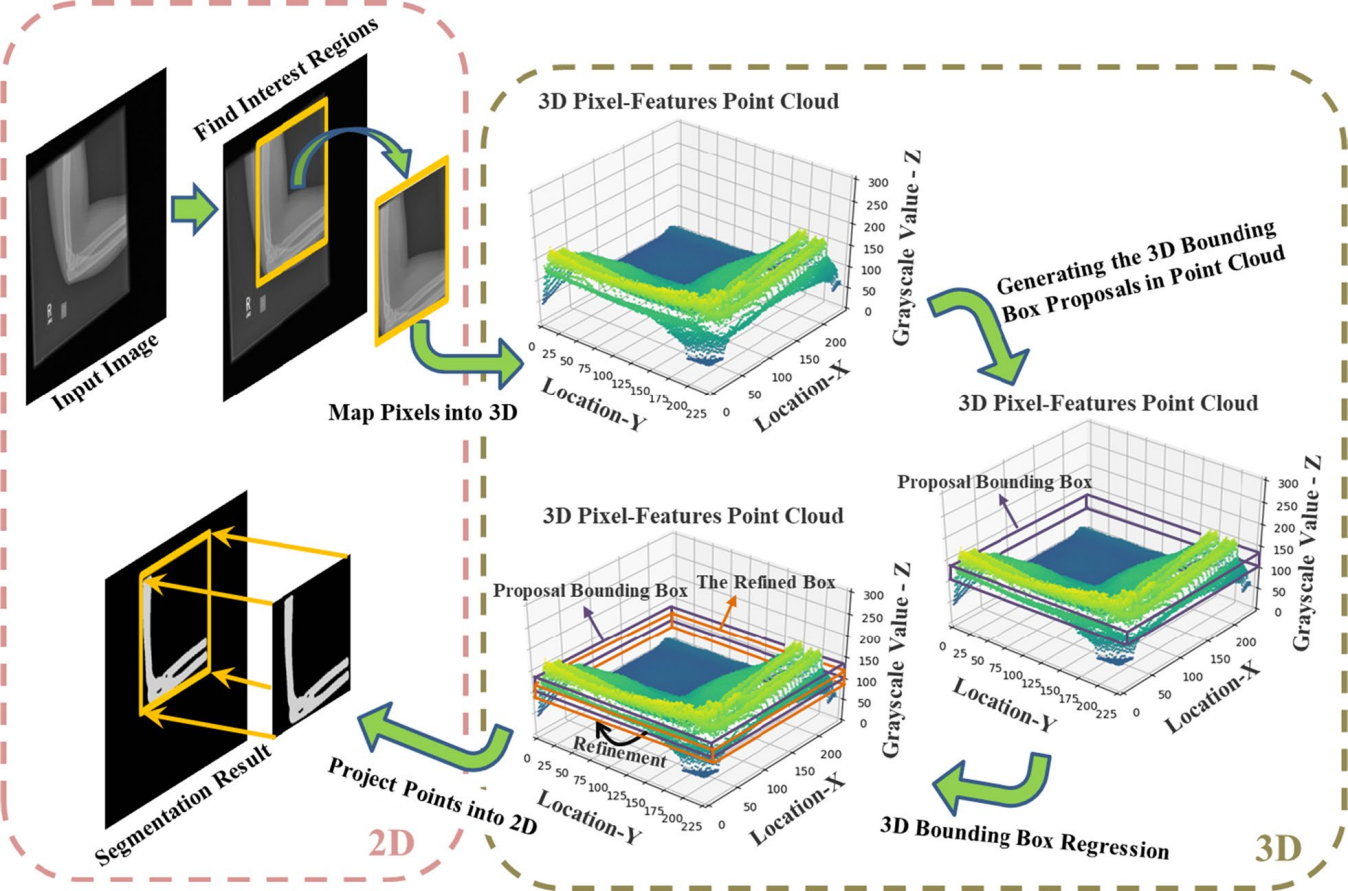
The pipeline of the proposed grayscale medical image segmentation method

**Fig. 4:**
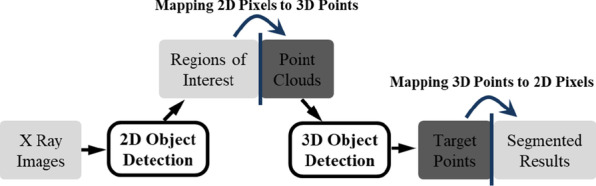
The implementation flow of the proposed method

The proposed image segmentation method maps each pixel in the medical grayscale image to 3D coordinates as the pixel-features point cloud, according to their positions and gray values. By acquisition of foreground points and their corresponding bounding box using 3D object detection method, we could achieve threshold values and the segmentation result of the corresponding grayscale image. The whole pipeline and the implementation flow of this method are shown in Figs. 3 and 4 respectively. Given a grayscale medical image, after (1) obtaining interest regions of associated segmentation objects in the image, (2) generating 3D bounding box proposals in point cloud and (3) the regression of their locations and scales, the refined boxes could be achieved. The projection of points in refined bounding box into the 2D image is the segmentation result.

#### Related work

According to the proposed strategy and above pipeline, object detections play the central roles at each block of our method. Many researches about 2D&3D object detection has raised ever and they could perform well especially those with deep learning.

The current mainstream 2D object detection methods based on deep learning could be generally classified into two-stage and one-stage methods [27]. With two-stage methods, proposal bounding boxes are generated firstly and the further refinement of proposals and confidences is obtained in the second stage [28]. While using the one-stage methods [29, 30], the location and the classification of object bounding boxes could be estimated directly without refinement which means one-stage methods are usually faster than two-stage ones but have lower object detection accuracy [31].

The widespread application of 3D geometric data spurs the development of 3D object detection and it could be categorized into monocular/stereo image-based, point cloud-based and multimodal fusion-based methods in terms of the modality of input data [32]. Due to point clouds are the most regular data which could be achieved with different sensors, enormous researches of point cloud-based methods have raised [33–35]. Among these method, different data format like raw point clouds or 3D voxel grids transformed from points could be feed into deep net architectures to find targets with bounding boxes and their classes [36].

#### Achievement of interest regions in image

**Fig. 5:**
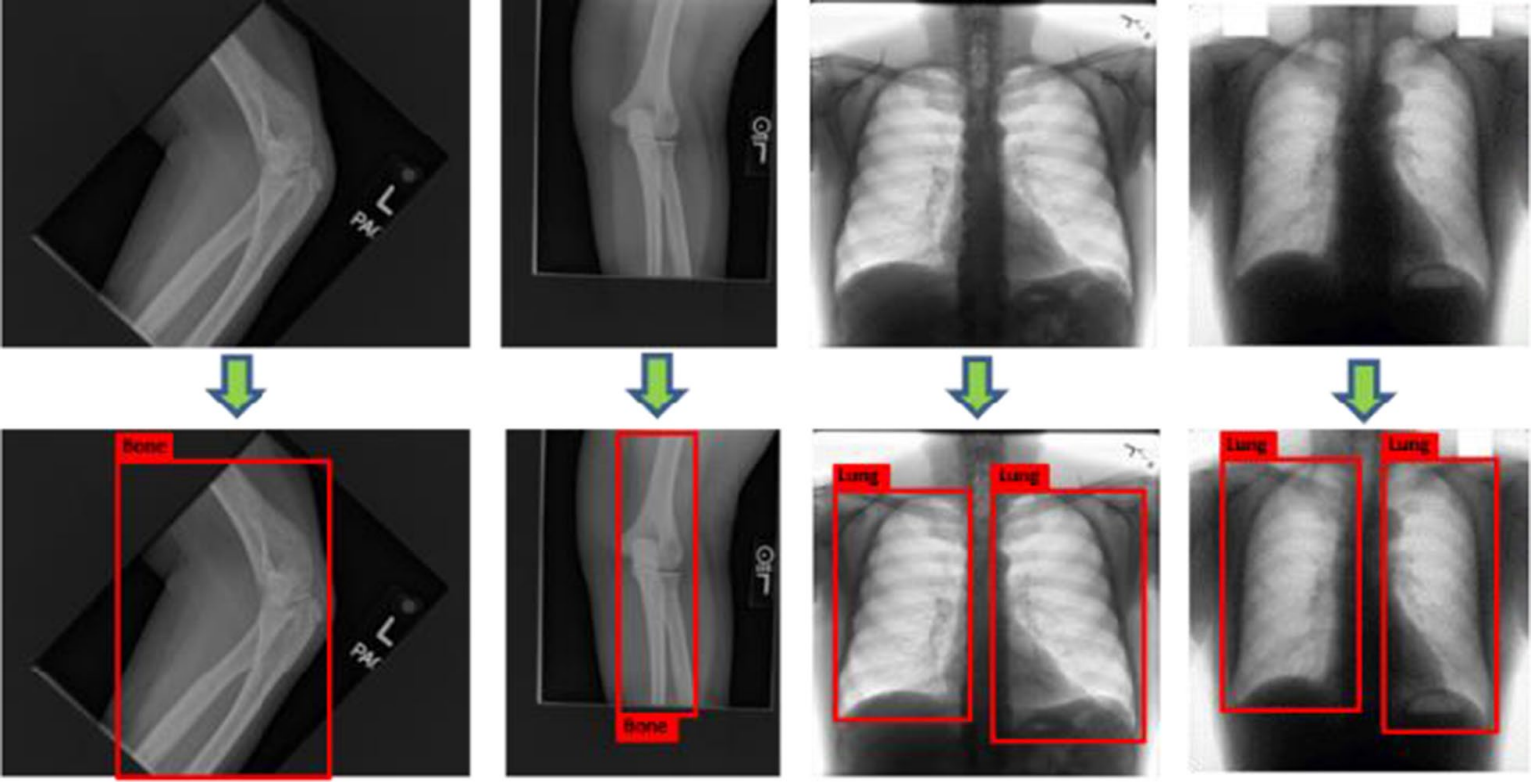
Achievement of interest regions in 2D images

In a medical grayscale image, pixels of the segmentation object always just take up a part of the entire image and there may exists noisy pixels with the same gray values in irrelevant regions. Therefore, 2D object detection is adopted as the pre-processing procedure to identify the specially interest regions with segmentation objects and reduce noisy pixels as shown in Fig. 5.

**Fig. 6:**
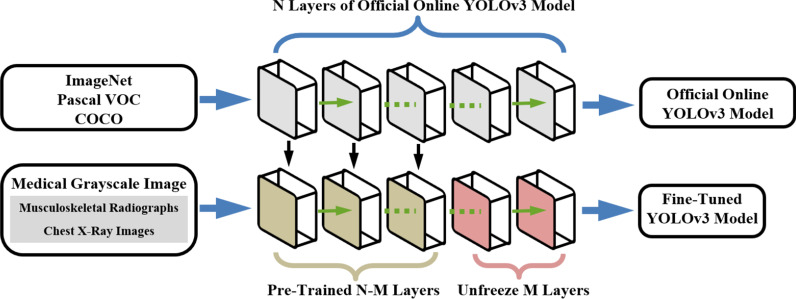
The proposed 2D object detection network with fine-tuning method

Compared with the accuracy, the proposed pre-processing procedure cares more about the detection speed, so we adopt the one-stage method YOLOv3 [37, 38] as the backbone network. And considering the scarcity of labeled medical grayscale images, we apply the fine tuning—a transfer learning method [31] to migrate most layers of the backbone model which was pretrained on ImageNet, Pascal VOC (Pattern analysis, statistical modeling and computational learning visual object classes) and MS COCO (Microsoft common objects in context) datasets [39, 40]. As Fig. 6. shown, with fine tuning method, we could freeze N–M layers of pre-trained model and only train the last M layers on local dataset. In order to retain the detection ability of pre-trained model as much as possible, and ensure the stability of the loss change during the training process, the proposed image segmentation pre-processing method only unfreeze the last 3 layers of pre-trained network for training.

#### Generation of proposal bounding box in pixel-features point cloud

The grayscale value of each pixel in interest regions represents their brightness [41]. Pixels compose the same tissues in particular image always share the grayscale value ranges and we could recognize them manually. All values range from 0 to 255 (Typically zero is taken to be black, and 255 is taken to be white). Darker pixels represent structures like soft tissues having less attenuation to the beam, while light ones represent structures like bones having high attenuation. Due to the lack of detailed gray values of pixels displayed on 2D images, it is hard to determinate their specific grayscale value ranges.

**Fig. 7:**
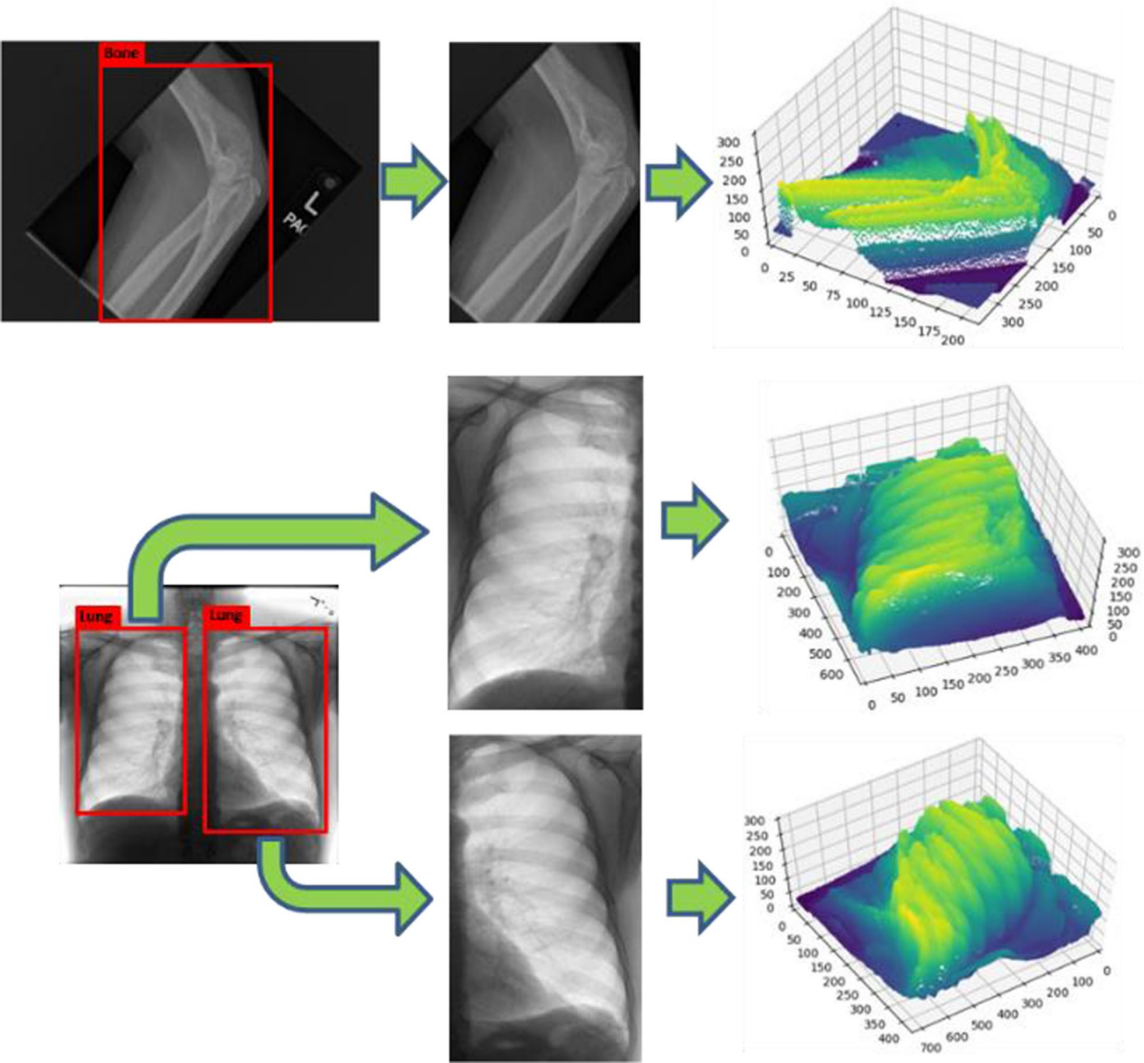
Turning pixels in interest regions into the pixel-features point cloud

Thus, we turn pixels in 2D interest regions into the 3D representations as Fig. 7 shown. In Fig. 7. the first two dimensions represent pixels locations and the third dimension represents their grayscale values. The 3D data could be considered as the pixel-features point cloud and it is distinct and intuitive to obtain points which represent pixels belong to the same tissues. This helps us translate the 2D image segmentation task into the 3D object detection with point cloud. We only need to determine locations and widths of 3D bounding boxes which contain the foreground points during the object detection. Then bottoms and tops of bounding boxes could represent the segmentation required threshold values for 2D images.

Inspired by two-stage 2D object detection methods, we present a novel two-stage 3D object detection method, which is operated on pixel-features point cloud. In the first stage of existing popular two-stage 2D object detection method, the proposal bounding boxes with its classification scores are generated with convolutional neural network and the refinements of those boxes are obtained in the following stage after the Non-Maximum Suppression (NMS). While in our proposed 3D object detection method, based on two-stage strategy, the proposal 3D bounding boxes with the classification scores of points inside them are estimated firstly and these proposals are refined with regression in second stage.

**Fig. 8:**
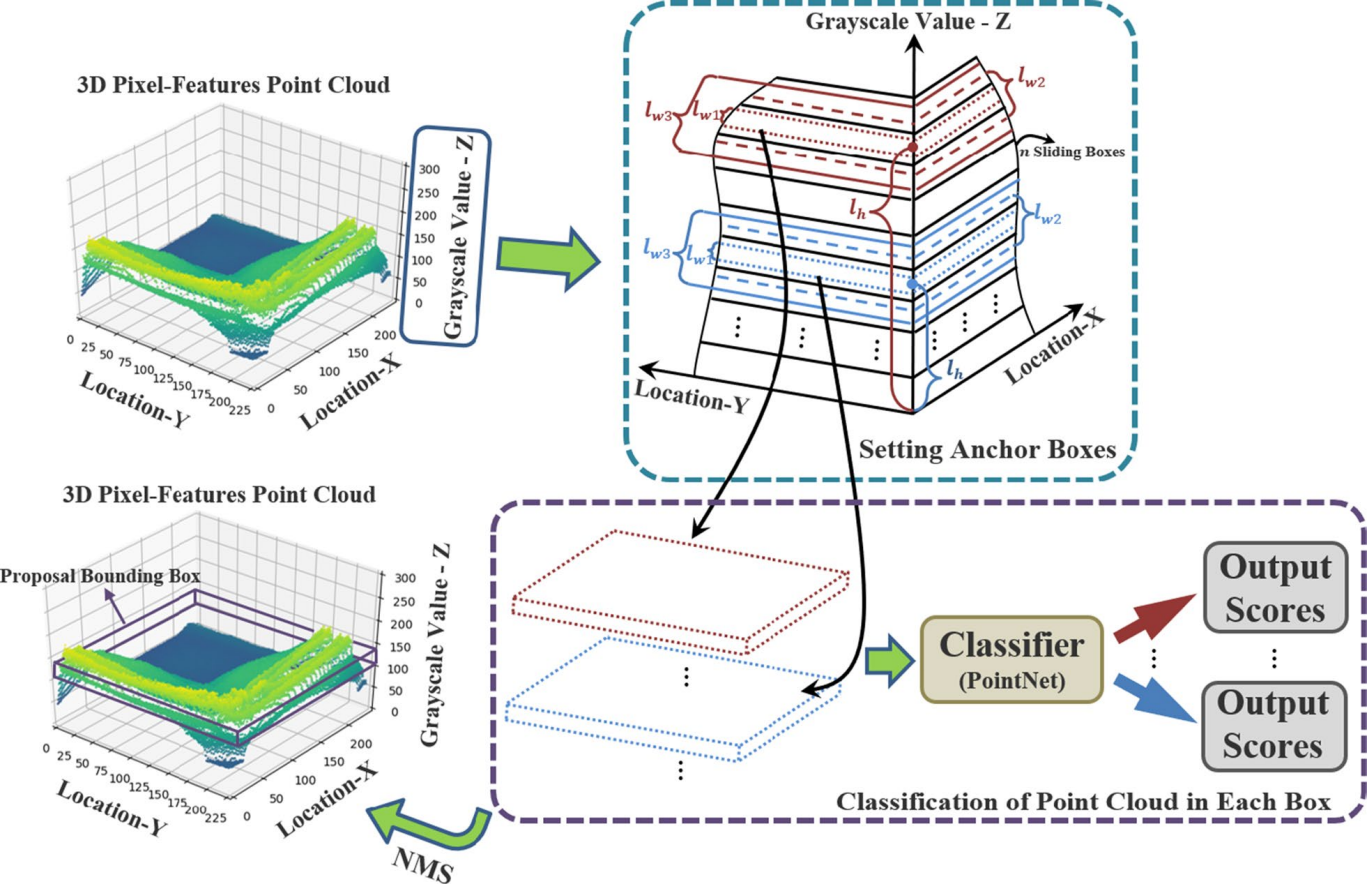
The generation of proposal bounding boxes in pixel-features point cloud

The generation of proposal bounding boxes in pixel-features point cloud has three modules. As shown in Fig. 8, These modules include localization of anchor boxes, classification of points inside boxes utilizing PointNet [36] as backbone network and Non-Maximum Suppression with 3D Intersection-over-Union (IoU).

##### Anchor boxes

Proposal bounding boxes generation takes the ${l}_{x}\times {l}_{y}\times 255$ point cloud representation as input where ${l}_{x}$ and ${l}_{y}$ respectively indicate the length and width of 2D interest region. In order to avoid high overlap rate of predict boxes and the low search efficiency using selective search as Region Convolutional Neural Network (RCNN) method, inspired by the Region Proposal Networks (RPN) in Faster RCNN, we apply the anchor boxes method for electing predict boxes.

To generate proposals, we slide a small network over the input by a shared 3D convolutional layer referred to RPN and Single Shot MultiBox Detector (SSD) method as Fig. 8 shown. At each sliding-box location, we could predict multiple proposals simultaneously, and we denote the maximum number of possible proposals as $k$. These proposals are parameterized relative to $k$ 3D anchor boxes. Each anchor is centered at its corresponding sliding box and is associated with a scale. Each anchor is defined with coordinates $({l}_{h},{l}_{w})$ where ${l}_{h}$ and ${l}_{w}$ represent its location and scale. We apply 3 scales by default, deciding $k=3$ anchors at each sliding box and $n\times k$ anchors in total.

##### Classification of point cloud

Anchor boxes with different scales share the same box-length ${l}_{x}$ and box-width ${l}_{y}$, and they are distinguished by their center locations and box-heights. In order to determine the proposal bounding box from numerous anchor boxes, we utilize the PointNet as our backbone network and apply the fine-tuning method for training our classification module.

The classification network in Fig. 8 indicates that raw point clouds are directly taken as the input and each point is processed independently at the initial stage. Due to point clouds could be easily applied rigid or affine transformations, input points are sorted into a canonical order with the first affine transformation by a mini-net (T-net) and moreover, after points features extraction with multi-layer perceptron (mlp), features from different points could also be aligned using another alignment network by feature transformation matrix. Then, the max pooling layer aggregates all points features extracted from the second mlp and outputs the global features. The final fully connected layers set the global feature as input and outputs $k$ scores for all the $k$ candidate classes.

**Fig. 9:**
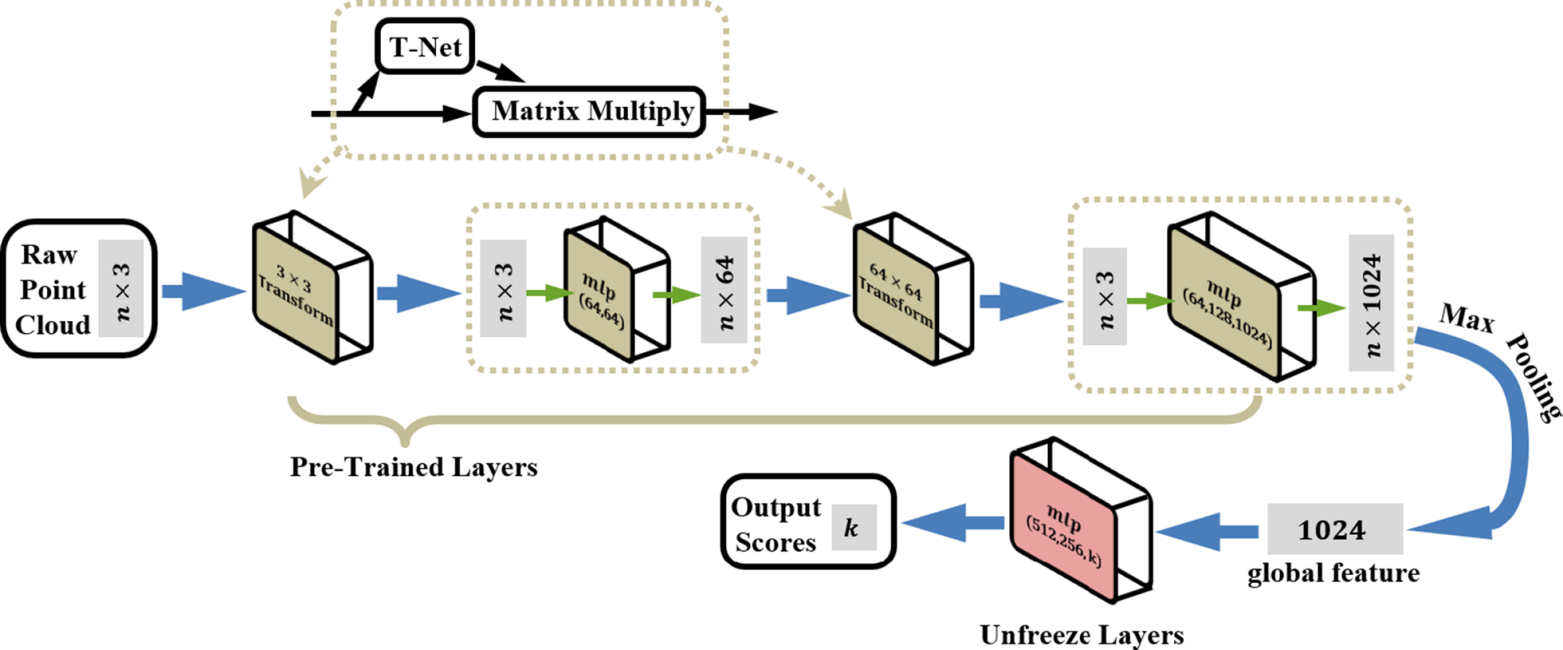
The proposed point cloud classification network with fine-tuning method

It should be noted that models-based point clouds datasets which mapped from grayscale medical images is scarce, thus we apply the fine-tuning method again. With the migration of PointNet model pretrained on ModelNet40 [42], we freeze most layers of the network except the final fully connected layers as shown in Fig. 9.

##### NMS with 3D IoU

**Fig. 10:**
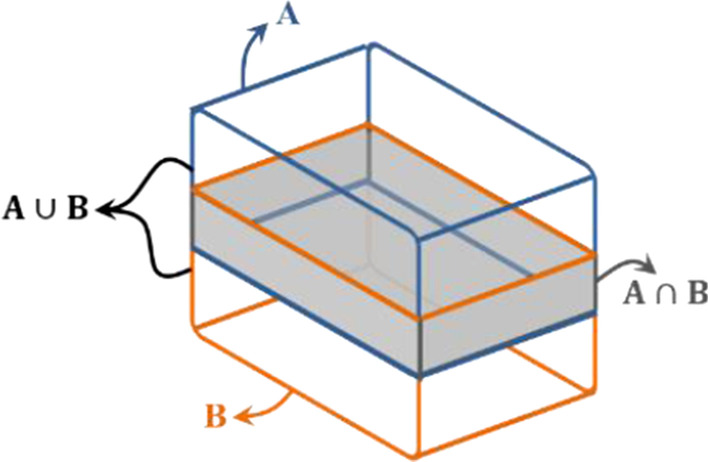
IoU computation for 3D. The intersection volume is highlighted in gray

After the above module, the classification results of point cloud in each anchor box could be achieved with scores. But as many 2D object detection method, there exists some repeated proposals of one object. They belong to the same candidate class and overlap with the local highest-score box. For reducing the redundancy, we adopt the non-maximum NMS on these proposals with 3D intersection over union (3D IoU). Different from the IoU computation for 2D based on the relationships of areas between box $A$ and $B$ [43], like Fig. 10 shows, volumes of two boxes are applied for 3D IoU calculation [44] which could be formulated as:

1 $$3{\text{D IoU}}\left( {A,B} \right) = \frac{{A_{v} \cap B_{v} }}{{A_{v} \cup B_{v} }} = \frac{{A_{v} \cap B_{v} }}{{\left| {A_{v} } \right| + \left| {B_{v} } \right| - A_{v} \cap B_{v} }}$$

Through the setting of 3D IoU threshold for NMS and ranking with classification scores, it remains only one box for each candidate class which could be considered as the proposal bounding box.

#### Refinement of proposal bounding box

Even though high classification scores of the proposal bounding boxes, the location and scale errors between them and ground truth exist. We train and implement a class-specific bounding box linear regression model to reduce errors and improve detection performance.

**Fig. 11:**
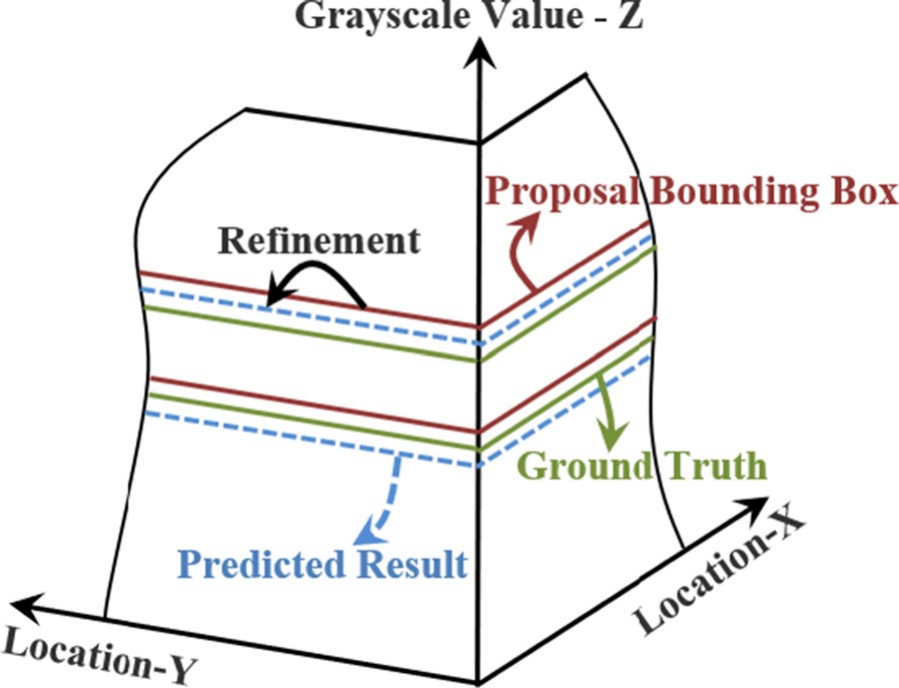
Refinement of proposal bounding box

On the assumption that we achieve one proposal bounding box ${P}^{i}$ and its nearby ground-truth box ${G}^{i}$ as shown in Fig. 11, where ${P}^{i}=({P}_{{l}_{h}}^{i},{P}_{{l}_{w}}^{i})$ specifies height ${l}_{h}$ of the center of proposal bounding box together with its width ${l}_{w}$. Meanwhile, the ground-truth bounding box ${G}^{i}$ is specified in the same way: ${G}^{i}=({G}_{{l}_{h}}^{i},{G}_{{l}_{w}}^{i})$. The goal of the bounding box regressor is to learn a transformation which could map each proposal bounding box $P$ to the ground-truth box $G$.

The transformation could be parameterized in terms of two functions ${d}_{{l}_{h}}(P)$ and ${d}_{{l}_{w}}(P)$. The first function specifies the translation of bounding box $P$’s center which is scale-invariant, while the second specifies the log-space translation of its width. By applying the transformation as following equations, an input proposal bounding box $P$ could be transformed into a predicted ground-truth box $\widehat{G}$.

2 $$\hat{G}_{{l_{h} }} = P_{{l_{w} }} \times d_{{l_{h} }} \left( P \right) + P_{{l_{h} }}$$

3 $$\hat{G}_{{l_{w} }} = P_{{l_{w} }} \times \exp \left( {d_{{l_{w} }} \left( P \right)} \right)$$

where $\mathrm{exp}$ is the natural exponential function.

Inspired by the 2D object detection, the bounding box regression of our method is performed on global features which is max pooled from PointNet model. Above two functions ${d}_{{l}_{h}}(P)$ and ${d}_{{l}_{w}}(P)$ could be modeled as linear functions of the global features of proposal bounding box $P$, denoted as ${f}_{mp}(P)$. Therefore, we have ${d}_{*}\left(P\right)={\mathrm{T}}_{*}\times {f}_{mp}(P)$, where $*$ represents ${l}_{h}$ or ${l}_{w}$, and ${\mathrm{T}}_{*}$ is a vector composed of learnable model parameters.

The transformation targets ${t}_{*}$ between proposal bounding box $P$ and the real ground-truth box $G$ could be defined as:

4 $$t_{{l_{h} }} = \frac{{G_{{l_{h} }} - P_{{l_{h} }} }}{{P_{{l_{w} }} }}$$

5 $$t_{{l_{w} }} = \log \left( {\frac{{G_{{l_{w} }} }}{{P_{{l_{w} }} }}} \right)$$

Thus, after setting the loss function and by optimizing the regularized least squares objective as following, we could learn ${\mathrm{T}}_{*}$ and achieve the transformation to refine the proposal bounding box.

6 $${\varvec{Loss}} = \mathop \sum \limits_{i}^{N} \left( {t_{*}^{i} - {\hat{\text{T}}}_{*} \times f_{mp} \left( {P^{i} } \right)} \right)^{2}$$

7 $${\text{T}}_{*} = {\text{argmin}}_{{{\hat{\text{T}}}_{*} }} {\varvec{Loss}} + \lambda {\hat{\text{T}}}_{*}^{2}$$

where $\mathrm{argmin}$ means ${\mathrm{T}}_{*}$ depends on the minimum of ${\varvec{L}}{\varvec{o}}{\varvec{s}}{\varvec{s}}$.

#### Obtaining segmentation results

As shown in Fig. 3, 3D bounding boxes in space could represent 3D positions range of pixel-feature points among them. Since 3D point cloud is mapped from pixels in 2D images according to positions and grayscale values, 3D bounding boxes could also present positions range and grayscale values range of 2D pixels corresponding to 3D points among boxes. After the refinement, 3D bounding boxes with accurate height and weight could be achieved. The top and the bottom of refined boxes represent the thresholding values for segmentation, while the front, back, left and right of boxes describe regions for segmentation. By remapping points among refined 3D bounding boxes to pixels in 2D images, these 2D pixels could compose segmentation results.

#### Training strategy

The proposed grayscale medical image segmentation method is based on 2D and 3D object detection models. Transfer learning and piecewise learning rate are applied for object detection models training. In the training of 2D object detection model, YOLOv3 which is trained on datasets including ImageNet, Pascal VOC and MS COCO is selected as the pretrained model. In the first stage, all but last 3 layers are frozen and the model is trained with prepared datasets including musculoskeletal and chest radiographs in the learning rate as 0.001 for 25 epochs. In the second stage, all layers of the network are unfrozen and they are trained in the learning rate as 0.0001 for 25 epochs. It takes 1.75 h to train the 2D object detection model. 3D object detection is composed of point cloud classifier and 3D bounding box regressor. In the training of point cloud classifier, PointNet which is trained on ModelNet40 is chosen as the pretrained model. In the first stage, all layers except last mlp module are frozen and the model is trained with point could datasets of bone and chest in the learning rate as 0.001 for 100 epochs. Then, all layers are unfrozen and the model is trained in the learning rate as 0.0001 for 100 epochs in the second stage. It takes 2.25 h to train the point cloud classifier; In the training of 3D bounding box regressor, due to the simple model and the requirement of bounding box refinement, the model is trained with one-stage strategy in a small learning rate as 0.0001 for 200 epochs and the training takes 0.25 h. Besides, Adam is selected as the optimizer for training of 2D object detection model and point cloud classifier, while Stochastic Gradient Descent is chosen as the optimizer for 3D bounding box regressor training.

### Performance assessment

In this study, we evaluate the segmentation performance by following four metrics: Dice similarity coefficient (DSC) scores [6], intersection over union (IoU), False negative (FN) and False positive (FP) [7]. Ranges of DSC and IoU are between 0 and 1, higher values of them and lower values of FN and FP indicate the higher accuracy. The calculation formula of DSC is defined as:

8 $${\text{DSC}} = \frac{{2\left| {T \cap G} \right|}}{\left| T \right| + \left| G \right|}$$

where $T$ is the detected region and $G$ is the ground truth region.

## Results

Our model is implemented with Pytorch [45] and its entire training process is performed on a computer with Windows 10 operating system, Intel Core i7 processor with 3.0 GHz, 64 GB of RAM and a single NVIDIA GPU (Quadro RTX 4000).

**Fig. 12:**
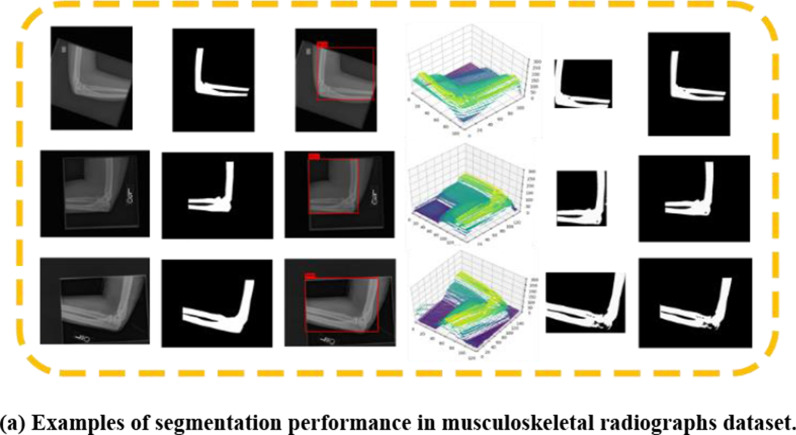

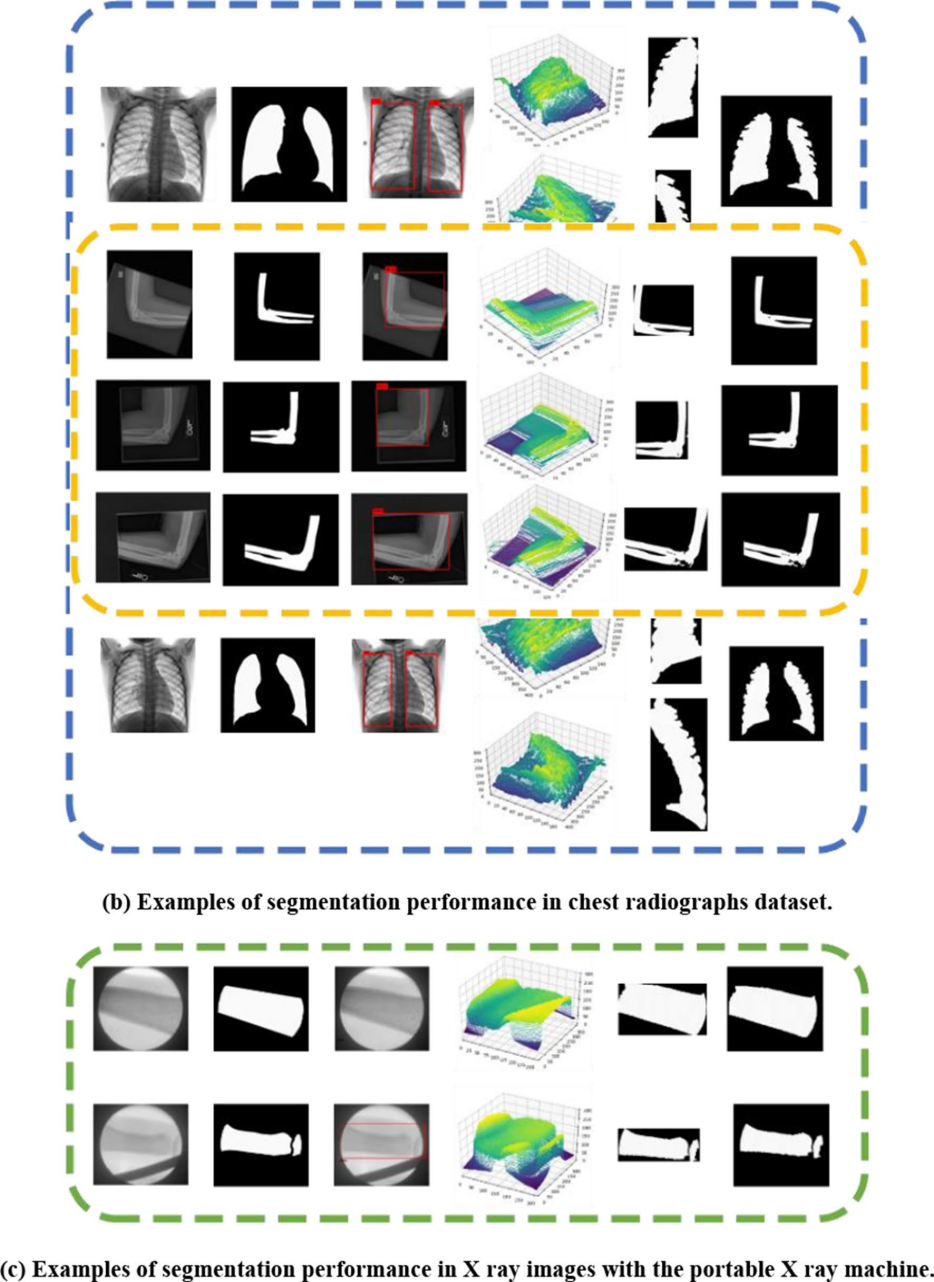
Segmentation results from different kinds of datasets. From the first to the last column are origin images, ground truth, achievements of interest regions, representations of pixel-feature point cloud, local segmentation results, and segmentation results in original image size, respectively

After training process, by applying the proposed method with the given grayscale medical images input and following the method pipeline as Fig. 3. shown, regions of target issues could be segmented. Each block in Fig. 12. presents several examples of segmentation performance from different kinds of datasets, as well as processing results after each stage, where white represents true positive pixels and black is for true negatives pixels. Moreover, according to evaluation criteria, Table 1 shows four metrics including IoU, DSC, FN and FP to assess the segmentation performance of images in different datasets.

**Table 1:** The values of evaluative metrics from experiments in different datasets

Datasets	IoU	DSC	FN	FP
Musculoskeletal radiographs	0.92	0.96	0.05	0.02
Chest radiographs	0.88	0.93	0.11	0.15
Images from X-ray machine	0.94	0.94	0.06	0.08

As shown in Fig. 12 and Table 1, we could obtain high IoU and DSC scores with satisficed segmentation results on different datasets. This indicates that based on the proposed method, 2D interest regions and 3D bounding boxes containing target pixel-features point cloud during the processing could be successfully achieved.

## Discussion

**Fig. 13:**
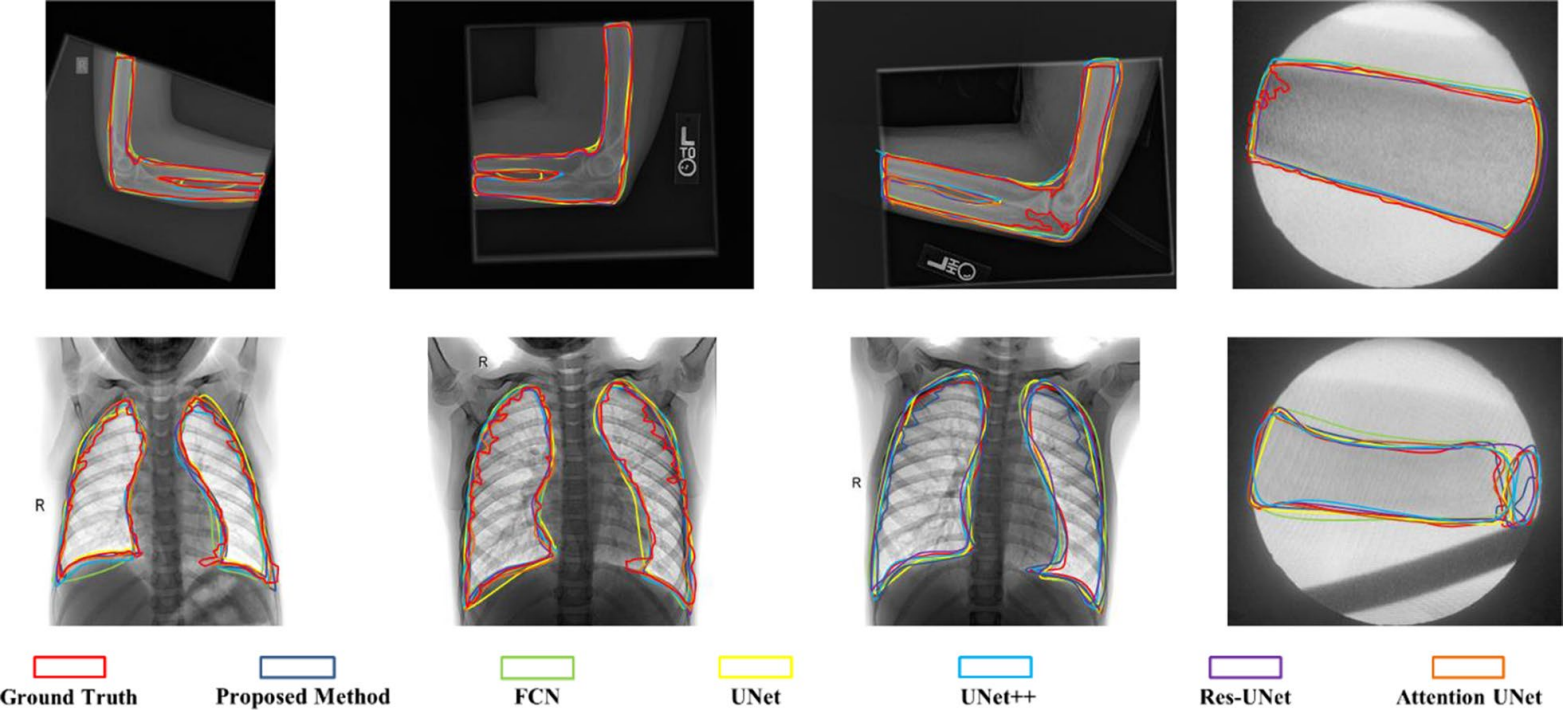
Comparisons of segmentation performance on each case in Fig. 12. with different methods

In this section, we compare the image segmentation performance of the proposed method with multiple famous and clinically performed well models. As well known, CNN based models are among the most successful and widely used for medical image processing. Besides the milestone FCN model, UNet built on top of the fully convolutional networks with a U-shaped architecture to capture context information, and based on it, Res-UNet [46] improved the segmentation results using residual blocks as the building block and UNet++ [47] enhanced segmentation quality of varying-size objects. Also, Attentiom UNet [48] achieved the better performance with the attention gate. We train these models in the same dataset as our proposed method and Table 2 presents the comparison results. Meanwhile, Fig. 13. shows results of each case in Fig. 12. with different methods by visualization. It indicates that compared with other models, our proposed approach improves the segmentation performance and it obtains the highest IoU scores of 0.92, 0.88 and 0.94 with three datasets respectively. In our approach, 2D and 3D object detection models could be both trained with transfer learning method which makes it possible to achieve a quite accurate image segmentation model with small training datasets. While other sematic segmentation methods may be sensitive to the scale of datasets because the pre-trained model could only help simplify the downsample training procedure, and the training of upsample still requires a number of datasets. This indicates that it is impossible to adapt them for every application task well because training data is scare especially in medical image field. Moreover, in grayscale images, grayscale values of pixels are important features to distinguish different objects, and the intuitive logic of grayscale image segmentation could be considered as the collection of pixels with similar grayscale values. So, the proposed image segmentation model which obtains the purpose ranges of grayscale values with 3D object detection have better explicability and segmentation effect.

**Table 2:** Comparison between segmentation performance (IoU) of the proposed approach with other methods

Datasets	Proposed	FCN	UNet	UNet++	Res-UNet	Attention Unet
Musculoskeletal radiographs	0.92	0.82	0.85	0.84	0.91	0.90
Chest radiographs	0.88	0.76	0.81	0.83	0.88	0.86
Images from X-ray machine	0.94	0.72	0.82	0.87	0.85	0.91

Under different medical imaging devices and environment in clinical, ranges of grayscale values of pixels which compose the same segmentation target in different medical images are always different. But our proposed method could settle this and we could obtain thresholding values (top and bottom of 3D bounding boxes) by mapping pixels in 2D images into 3D point clouds and adopting 3D object detection with features of pixels.

## Conclusions

In this paper, we present a new grayscale medical image segmentation method with object detection models. In this method, 2D object detection model is applied to achieve interest regions of segmentation objects. After mapping 2D pixels in interest regions to 3D point cloud according to their positions and grayscale values, 3D object detection model is adopted to obtain bounding boxes containing target pixels-feature points. After projecting these points to 2D images, they could composite segmentation results. Experiments results prove the better effectiveness and accuracy of our method than the other compared models. In clinical applications, more than improving segmenting performance with bone and chest X-ray images, the proposed segmentation method could also be carried over for other kinds of grayscale medical images in diagnosis efficiently and conveniently. This is because two object detection models could be trained separately with little labeling cost based on transfer learning method. Besides, pretrained models for both 2D and 3D object detection in our method could be changed and upgraded flexibly for further accurate segmentation results.

## Data Availability

Musculoskeletal radiographs and chest radiographs which support our research are available from Stanford ML Group. But restrictions apply to the availability of these data, which were used under license for the current study, and so are not publicly available. Data are however available from the authors upon reasonable and with permission of Stanford ML Group. While phalanx and forearm X-ray images are available only upon request by emailing authors due to the ethical restrictions on sharing these data which could contain potentially sensitive information of patients.

## Abbreviations

CNN(s): Convolutional neural network(s)
FCN: Fully convolutional networks
MURA: Musculoskeletal radiographs
LERA: Lower extremity radiographs
CheXpert: Chest radiography
GT: Ground truth
YOLO: You only look once
PASCAL VOC: Pattern analysis, statistical modeling and computational learning visual object classes
MS COCO: Microsoft common objects in context
NMS: Non-maximum suppression
IoU: Intersection-over-Union
RCNN: Region convolutional neural network
RPN: Region proposal networks
SSD: Single shot multiBox detector
mlp: Multi-layer perceptron
DSC: Dice similarity coefficient
FN: False negative
FP: False positive
